# Preface

**DOI:** 10.1098/rstb.2009.0030

**Published:** 2009-07-27

**Authors:** Brian Heap

**Affiliations:** St Edmund's College, Cambridge CB3 0BN, UK

Plastics, the environment and human health featured prominently in an international seminar on Planetary Emergencies held at Erice, Sicily in August 2006. Enormous advantages have accrued from the industrial development and application of plastics, but whereas plastics production has increased from 0.5 to 260 million tonnes p.a. since 1950 the amount regenerated is relatively small, so that the gap between production and regeneration continues to expand. Plastics litter has reached a figure of approximately 10 per cent of municipal waste mass worldwide, material that has a longevity measured in hundreds of years.

Among the steps taken so far, biodegradable plastics have proved an important advance yet not all are destroyed completely in natural environments, raising the question of the definition of biodegradable. Close attention has been given to risks associated with additives that improve the properties of plastics yet leak into the environment, food and beverages. Plastic piping used to deliver potable water is now widespread, but phthalate additives in a wide range of flexible plastics, industrial solvents, personal care products and medical devices can show anti-androgenic properties at certain concentrations. Bisphenol A (BPA), a major additive in polycarbonate plastic for lining metal cans and many other products, can reveal oestrogen-mimicking properties.

I, along with several in the Erice audience, had accepted that leach was below the levels of toxicity of concern for humans, other vertebrates or invertebrates. However, independent laboratory work in animals treated with BPA suggested effects on body weight and hormonal parameters resembling those found in diabetes and obesity. Phthalates and BPA have been found in pregnant women and foetuses, and many in the USA have phthalates in their urine. So the big question is—does it matter? Has the tolerable daily intake of additives been exceeded, affecting human health, wildlife and the marine environment?

Some of the Erice papers caused us to ask whether sufficient attention was being given to the discovery of alternative additives that would, for example, avoid any association with endocrine disruption. Was there a scientific consensus about risks associated with the rising accumulation, deposition and interaction of multiple forms of plastics and leached additives in the environment? Could a product-recycling loop be developed that effectively reduced the plastics burden on humans and the environment? Could an international partnership be forged between scientists, policy-makers and industry to arrive at new solutions (as in the displacement of CFCs)?

Such questions were believed to be of sufficient scientific and public merit that a themed issue of peer-reviewed papers has been assembled by guest editors closely involved in the subject. The papers, some of which were aired at Erice, address the implications of our dependency on plastics in today's consumer society. They aim to inform and update the debate about plastics and promote further studies relevant to the assessment and management of risk, if it exists.

